# Central and Peripheral GABA_**A**_ Receptor Regulation of the Heart Rate Depends on the Conscious State of the Animal

**DOI:** 10.1155/2011/578273

**Published:** 2011-11-17

**Authors:** Bo Hjorth Bentzen, Morten Grunnet

**Affiliations:** NeuroSearch A/S, Pederstrupvej 93, 2750 Ballerup, Denmark

## Abstract

Intuitively one might expect that activation of GABAergic inhibitory neurons results in bradycardia. In conscious animals the opposite effect is however observed. GABAergic neurons in nucleus ambiguus hold the ability to control the activity of the parasympathetic vagus nerve that innervates the heart. Upon GABA activation the vagus nerve will be inhibited leaving less parasympathetic impact on the heart. The picture is however blurred in the presence of anaesthesia where both the concentration and type of anaesthetics can result in different effects on the cardiovascular system. This paper reviews cardiovascular outcomes of GABA activation and includes own experiments on anaesthetized animals and isolated hearts. In conclusion, the impact of changes in GABAergic input is very difficult to predict in these settings, emphasizing the need for experiments performed in conscious animals when aiming at determining the cardiovascular effects of compounds acting on GABAergic neurons.

## 1. Introduction

Gamma-aminobutyric acid (GABA)ergic neurons are the major contributor to neuronal inhibition in the brain. The activity of GABAergic interneurons has profound impact on spike timing control, neuronal rhythms, and the activity pattern of neuronal circuits. The correct tonus for these neurons are therefore critical for a number of different conditions such as attention, perception, consciousness, working memory, sensorimotor processing, and schizophrenia [1–3]. Activation of GABAergic neurons is also applied for myorelaxation, anxiolytic treatment, sedation, and anaesthetics [4–6]. In addition to CNS effects GABAergic neurons also have a significant impact on the cardiovascular system. From this perspective GABAergic neurons can affect both electrical and hemodynamic parameters. CNS-controlled chronotropic effects on the heart can happen via effects on nucleus ambiguus that will subsequently affect the vagal tonus and thereby heart rate [7]. Additionally it has been suggested that GABA could have a direct effect on cardiac tissue [8]. In addition to direct or indirect effect on cardiac electrical parameters GABAergic input will also affect blood vessels and thereby participate in the control of vascular tonus and blood pressure, which reflexively has effects on heart rate [9].

GABA receptors come in two different families: Ionotropic GABA_A_ receptors and metabotropic GABA_B_ receptors. A description of the G-protein-coupled GABA_B_ receptors is beyond the scope of the present paper. 

GABA_A_ receptors are ligand-gated ion channels with chloride conductance. The functional channel is typically heteropentameric in structure even though homomeric *ρ*1, 2, and 3 receptors have been described [10]. A number of different subunits exist, and the exact subunit composition will determine the electrophysiological properties of the channel, thereby ultimately the phenotypic output in an *in vivo* situation [11]. The majority of GABA_A_ receptors contain *α*, *β*, and *γ* subunits but *δ*, *ε*, *θ*, and *ρ* can also be present [12]. Ligand binding to GABA_A_ receptor has been intensively studied, and detailed information is available. The GABA binding site is located at the interface between the *α* and the *β* subunit, and a number of important amino acids in the binding pocket have been identified [13]. The effect of positive allosteric modulators is well described for GABA_A_ receptors exemplified by benzodiazepine. The binding site is here located between the *α*-*γ* interface [14]. Antagonist are considered to bind in a pocket that is partly overlapping with the agonist site, with the difference being that antagonist can extend further into the solvent accessible cavity [15]. Pore blockers, in the form of picrotoxin, are also valuable experimental tools for addressing the effects of GABA_A_ receptors, albeit this toxin has no therapeutic use due to induction of convulsions. 

The present paper gives an overview of the known literature of GABAergic effects on the cardiovascular system with emphasis on the cardiac vagal neurons because of their major importance in controlling the chronotropic state of the heart. Supportive information will be given for experiments performed on isolated hearts and anaesthetized *in vivo* experiments under influence of different concentrations of isoflurane.

## 2. Parasympathetic Control of Heart Rate

**Figure 1:**
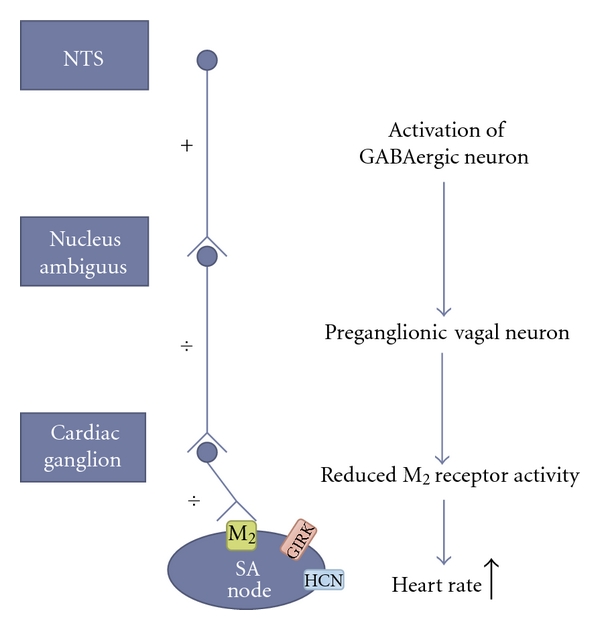
Model of how activation of GABAergic input to the cardiac vagal neurons increases heart rate. GABAergic neurons from the nucleus tractus solitarius inhibits the preganglionic cardiac vagal neurons, which leads to reduced postganglionic vagal input to the heart. Consequently, the muscarinic acetylcholine receptor (M_2_) activity is reduced. Because the G_i _ protein no longer inhibits the production of cAMP by the adenylyl cyclase, HCN channel activity is increased. In addition the G protein-coupled inwardly rectifying potassium channel (GIRK) is no longer activated. Together this will cause the heart rate to increase.

Cardiac cells hold the ability to initiate action potentials. The sinoatrial node has the highest automaticity, and consequently the normal heart beat originates from here. The heart activity is however regulated by the intrinsic cardiac neurons, by hormones and by the sympathetic and parasympathetic branches of the autonomic nervous system [16]. The parasympathetic cardioinhibitory effects are opposed by the facilitatory sympathetic effects. In both animals and humans the parasympathetic tonus dominates over the sympathetic at rest [17–19]. Cardiac preganglionic neurons whose cell bodies are primarily found in the nucleus ambiguus and some in the dorsal motor nucleus of the vagus are responsible for this tonic control of heart rate. They are also important for the reflex and respiratory regulation of heart rate seen in response to baroreceptor activation and inspiration [20]. Via the vagal nerve, the axons from these nuclei reach the intrinsic cardiac ganglia located in the fat pads adjacent to the right atrium [16]. From here postganglionic neurons innervate the sinoatrial node, ultimately leading to activation of cholinergic G-protein-coupled receptors (M_2_) and heart rate reduction. This happens primarily through activation of the acetylcholine-activated K^+^ channel GIRK, but also by M_2_ receptor-induced inhibition of the adenylyl cyclase, which ultimately leads to reduced HCN channel opening probability [21, 22] (Figure 1). The preganglionic cardiac vagal neurons do not hold any intrinsic pacemaker activity and are consequently intrinsically silent [23]. Therefore, they must rely on synaptic input mediated by both ionotropic and G-protein-coupled receptors for controlling their firing [24–26]. Excitatory inputs to the cardiac vagal neurons include both glutamatergic inputs predominantly from the nucleus tractus solitarius, and pre- and postsynaptic cholinergic nicotine receptors that can excite the cardiac vagal neurons (for review see [27]). With respect to the inhibitory GABAergic input to the cardiac vagal preganglionic neurons, it was found that inhibition of GABA_A_ receptor activity by microinjection of bicuculline into nucleus ambiguus resulted in dose-related reduction of heart rate and that the effect was reversed by the GABA_A_ receptor agonist muscimol [7]. This demonstrates the importance of GABAergic input to the nucleus ambiguus in setting the tonic heart rate level. The phasic inhibition of cardiac vagal neurons by activation of GABA_A_ receptors in the nucleus ambiguus is important for the respiratory sinus arrhythmia where the heart rate slows during inspiration and for the baroreflex where changes in arterial pressure reflexively cause homeostatic changes in heart rate [28].

Upon synaptic release of GABA multiple types of postsynaptic GABAergic receptors on the parasympathetic cardiac neurons in the nucleus ambiguus are activated. *In vitro* electrophysiological studies found that the phasic inhibitory currents observed after release of GABA could be blocked by gabazine whereas gabazine-insensitive but picrotoxin-sensitive receptors were responsible for the tonic inhibitory synaptic currents [30]. This phenomenon is well known in other brain areas [31]. When the tonic currents were blocked the membrane potential depolarized and increased the firing activity of the cardiac vagal neurons. How the tonic GABA current is activated in the nucleus ambiguus is unknown, but a role for the GAT-1 GABA transporter is unlikely as inhibition of this did not augment the magnitude of the tonic GABAergic current in the vagal neurons [30]. Other mechanisms such as spontaneous opening of constitutive active GABA channels could be responsible for the tonic GABAergic current. Bouairi et al. further demonstrated that application of the benzodiazepine, flunitrazepam, increased the decay time of the phasic IPSC and augmented the tonic current [30]. This would decrease the input resistance of the neuron and serve as a “sink” for any excitatory inputs [30]. These findings also provide a hint to the molecular composition of the cardiac vagal neuron GABA_A_ receptor. The positive allosteric modulation by benzodiazepines requires the presence of a *γ*-subunit, with the binding site located at the *α*-*γ* interface [14]. In the brain stem *α*1 and *α*3 are more strongly expressed than *α*2, *α*4, *α*5, and *α*6 (for review see [32]). However, the molecular composition of the GABA_A_ receptors in the cardiac vagal neurons needs to be determined. 

The sources of GABAergic neurotransmission to the cardiac vagal neurons in the nucleus ambiguus have been investigated. Both with respect to the tonic and phasic inhibitory GABAergic inputs they have been suggested to in part originate from the nucleus tractus solitaries (NTS) [33]. Electrical stimulation of the NTS produces a GABAergic current in the cardiac vagal neurons that can be blocked by bicuculline [33]. An excitatory monosynaptic glutamatergic pathway from NTS that activates NMDA and non-NMDA postsynaptic receptors in cardiac vagal neurons has also been identified [26]. By photo-uncaging glutamate in the near vicinity of GABAergic neurons and simultaneously recording from cardiac vagal neurons, Frank et al. could identify and map GABAergic neurons projecting to the cardiac vagal neurons in the nucleus ambiguus. Using this technique they identified areas in the nucleus tractus solitarius and in the close proximity to the nucleus ambiguus that when stimulated by uncaging of glutamate evoked a GABAergic inhibitory response in the cardiac vagal neurons [34].

The nucleus tractus solitarius is important for integrating the autonomic nervous system functions and so also for cardiovascular and respiratory regulation and reflexes. The NTS receives afferent input from the cranial nerves and hence information from a variety of organs and visceral regions, including sensory information from chemoreceptors and arterial baroreceptors [35, 36]. These sensory information is important for the baroreflex, where increase in blood pressure causes afferent baroreflex activity, that activates neurons in the NTS and possibly via the excitatory glutamatergic pathway evokes an increase in the cardiac vagal neuron activity and a compensatory decrease in heart rate (for review see [20, 37]). Respiration also influences the cardiac vagal neuron output through reflex mechanisms. One of these reflex mechanisms is the respiratory sinus arrhythmia, which describes the changes in the heart rate with respiration. From ECG recordings this can be observed as a shortening of the R-R interval (increased heart rate) during inspiration and a prolongation during expiration. This phenomenon has been suggested to save cardiac energy by effectively reducing the number of heartbeats during expiration, providing an efficient ventilation/perfusion matching [38]. Respiratory sinus arrhythmia is primarily mediated by altering the firing pattern of the cardiac vagal neurons. They are silent during inspiration and active during expiration. This rhythm is not achieved by changes in excitatory pathways projecting to the cardiac vagal neurons but rather by the activation of inhibitory pathways during inspiration which increases the GABAergic and glycinergic input to the cardiac vagal neurons, thereby lowering the vagal tone on the heart during inspiration [20, 39]. Respiratory sinus arrhythmia which has been observed in many different mammals is blunted, diminished or even reverted in anaesthetised animals depending on the anaesthetics used [40]. This demonstrates the importance of understanding how general anaesthetics work, especially when investigating the parasympathetic control of heart rate or when novel compounds are investigated for cardiovascular safety liability.

## 3. Effects of General Anaesthetics on the Heart

Many general anaesthetics have been found to affect cardiovascular reflexes by interfering with the cardioinhibitory vagal neurons in the nucleus ambiguus. Pentobarbital is often used for induction of anaesthesia and is known to cause respiratory depression, blunted baroreflex, and increased heart rate [41]. These effects are primarily related to the pentobarbital induced potentiation of the spontaneous postsynaptic inhibitory currents in the cardiac vagal neurons, whereby the cardioinhibitory parasympathetic input to the heart is decreased, and heart rate is increased [42]. Similarly, propofol, which is also known to potentiate GABA_A_ currents, augments the GABAergic input to the cardiac vagal neurons by increasing both phasic and tonic GABA_A_ receptor currents. This evokes an increase in heart rate [43, 44]. At supratherapeutic propofol concentrations inhibition of the GABAergic neurotransmission to the nucleus ambiguus is observed with a subsequent reduction in heart rate [44]. Isoflurane is known to decrease blood pressure, evoke respiratory inhibition, and to cause variable changes in heart rate depending on the depth of anaesthesia [45, 46]. Furthermore, studies have demonstrated that the baroreceptor reflex was not depressed significantly until 2.6% isoflurane (2X minimum alveolar concentration (MAC)) [47]. Other reflexes such as the respiratory sinus arrhythmia are however compromised during isoflurane exposure at clinically relevant concentrations [48]. Isoflurane increases heterologously expressed GABA_A_ receptor currents with the effect peaking around 1 MAC. The concentration response curve is bell shaped, and eventually isoflurane produces an inhibition of the GABA_A_ steady-state current as the isoflurane concentration increases. In order to lower the concentration of isoflurane needed to achieve the desired anaesthetic depth, the volatile anaesthetic is often supplemented with nitrous oxide (N_2_O). From electrophysiological recordings of heterologously expressed GABA_A_ receptors addition of N_2_O results in an augmentation of the potentiating effects of isoflurane [49]. The enhancement of GABA_A_ receptor currents by isoflurane results in prolonged inhibitory postsynaptic currents, increased Cl^−^ influx, and reduced excitability. In addition, recordings from cardiac vagal neurons in the nucleus ambiguus demonstrated that isoflurane also enhanced the tonic GABAergic current [43]. Taken together this augmentation of the GABAergic input to the cardiac vagal neurons would result in reduced vagal excitability. This would shift the parasympathetic/sympathetic balance resulting in tachycardia. However, Wang also measured a reduced frequency of GABAergic IPSC after exposure to isoflurane, which would lessen the inhibition of the parasympathetic output from the vagal nerve, resulting in less tachycardia [43, 50]. Other anaesthetics do not induce tachycardia. The synthetic opiate fentanyl produces bradycardia partly via inhibition of the GABAergic pathways to the cardiac vagal neurons in the nucleus ambiguus [51].

With respect to cardiovascular safety pharmacology, knowledge about the profound effect of anaesthetics on the heart and vasculature is important when designing and interpreting studies of novel compounds and their possible detrimental effects on the cardiovascular system. Considering that GABA is the main inhibitory neurotransmitter in the central nervous system and the importance of the GABAergic system in determining the firing activity of the vagal cardiac neurons, it is maybe of no surprise that drugs modulating GABA_A_ receptor activity will influence cardiac parameters. However, the outcome of such modulations can be hard to interpret and predict due to the complexity of these integrative systems and reflexes. 

## 4. Cardiovascular Effects of Positive Allosteric Modulators of GABA_A_ Receptors

Benzodiazepines are positive allosteric modulators of GABA_A_ receptors. The cardiovascular effects of benzodiazepines have been investigated in both animals and humans. Animal studies have found that benzodiazepines result in lowered blood pressure and variable effects on heart rate. Findings of reduced heart rates in anaesthetized animals were explained by reduction of sympathetic outflow [52, 53]. In anaesthetised rats two benzodiazepine tested produced tachycardia, and this effect was attenuated by pretreatment with atropine [54], suggesting an important role of the parasympathetic nervous system. This role is also stressed by a study demonstrating that application of the benzodiazepine, flunitrazepam, increased the GABAergic input to the cardiac vagal neurons, which lowered their excitability and consequently reduced the parasympathetic outflow to the heart [30]. Differences in species, the anaesthetics, and the depth of anaesthesia used might help to explain these varying effects on heart rate. Because of the profound effects of general anaesthetics on cardiac regulation, the effects of positive allosteric modulators of GABA_A_ receptors on conscious animals are important. In conscious trained dogs low doses of diazepam and bromazepam (p.o.) had no influence on heart rate, but a rapid onset positive chronotropic effect was observed at higher doses (10 mg/kg p.o.). The onset was rapid and could not be reverted by the beta-adrenoceptor blocking agents, indicating that the rapid heart rate was not solely a result of increased sympathetic outflow to the heart [55]. Using radiotelemetry devices diazepam (6 mg/kg i.p.) was found to increase the heart rate in conscious rats [56]. Using a similar setup another GABA_A_ receptor potentiator JM-1232 was also found to increase heart rate, and this effect was prevented by pretreatment with atropine or propranolol. This indicates an involvement of both branches of the autonomic nervous system. The authors suggest that the tachycardia could therefore be a consequence of GABAergic inhibition of the vagal nerve output and/or baroreflex activation due to hypotension [57]. 

In general the cardiovascular effects of benzodiazepines in humans, at clinically relevant doses, are mild [58]. However, during i.v. infusion, or with overdose, benzodiazepines may cause hypotension and respiratory depression [59, 60]. Short acting, fast on-set benzodiazepines such as midazolam are often used as premedication before surgical interventions. When midazolam is injected i.v. it produces a rapid drop in blood pressure and an increase in heart rate [59], comparable to what is seen in conscious animals. A study conducted to evaluate the influence of benzodiazepines on the autonomic neurocardiac regulation in humans found a similar rapid increase in resting heart rate and a concomitant reduction in vagal tone, assessed by changes in heart rate variability. A role for a baroreflex-induced tachycardia was ruled out as no significant fall in blood pressure was recorded [61]. 

**Figure 2:**
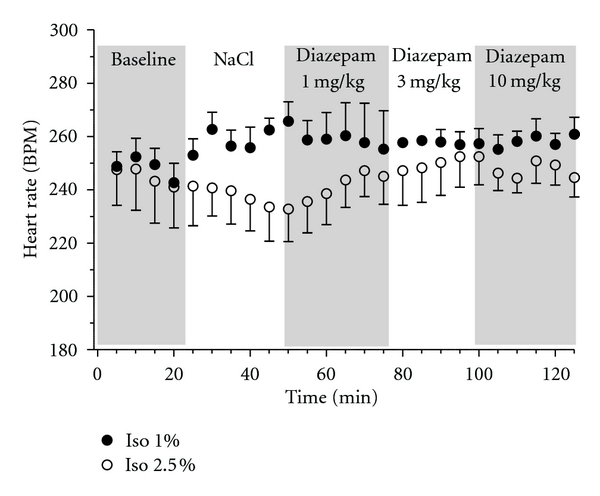
Effect of diazepam on heart rate in artificially ventilated (1% isoflurane, filled circles; 2.5% isoflurane, open circles; O_2_ : N_2_O 1 : 1) female guinea pigs (538 ± 27 g). The animals were placed on heated mats and the temperature was monitored and kept constant at 37 ± 1°C throughout the experiment. Electrocardiographic (ECG) recordings were obtained using 2 electrodes placed in the subcutaneous layer of the forelimbs (left and right), and 1 electrode placed in the subcutaneous layer of the left hind limb. ECG recordings were analysed using Chart ADinstrument software and Graphpad Prism 5. A stabilization period of minimum 20 min was performed, followed by NaCl 0.9% i.p. Subsequently the animals were injected intraperitoneally every 25th min with increasing concentrations of diazepam (1, 3 and 10 mg/kg i.p.).

Because the cardiac action potential in guinea pigs resembles more closely the human cardiac action potential as compared to other rodents, especially with respect to the repolarizing currents, guinea pigs are a preferred animal model for initial screening for cardiovascular safety liabilities [62]. In guinea pigs artificially ventilated with isoflurane (1% or 2.5%, O_2_ : N_2_O 1 : 1) administration of diazepam (1, 3 & 10 mg/kg i.p.) produced no significant changes in heart rate as compared to saline injection (Figure 2). No significant changes in heart rate corrected QT-interval, PR-interval, or temperature were observed (data not shown). 

The lack of effect of diazepam in the guinea pig on heart rate as compared to rat could be a species- or dosage-dependent phenomenon, but it could also stem from the use and depth of anaesthesia which might already augment the GABAergic input to the cardiac vagal neurons to such an extent that further potentiation of GABA_A_ receptors would not cause any vagolytic effect. In order to circumvent this we will establish an *in vivo *setup using radiotelemetry implants in order to investigate the *in vivo* effects of positive allosteric modulators of GABA_A_ receptors in conscious guinea pigs. This will also allow for simultaneous recordings of ECG, blood pressure, temperature, and locomotor activity. Such experimental condition will optimize the amount of data that can be extracted from a single experiment and aid in interpretation of drug-induced cardiovascular effects.

## 5. Effect of GABA on the Isolated Heart

In addition to the central mediated GABA_A_ receptor effects on the heart, studies have also suggested the presence of GABA_A_ receptors in the heart. One study found mRNA expression encoding the GABA_A_ receptor *ε* subunit in the human cardiac conduction study [63] and in mouse GABA_A_ receptor protein was detected in the heart [64]. GABA has been found in the guinea pig heart using [^3^H]-GABA, especially in the area of the SA node and in the intrinsic cardiac ganglion [8, 65]. It appears that there is no direct GABAergic pathway connecting the nervous system to the heart. Yet, GABA might exert its effect on the intrinsic cardiac neurons, where it appears to play indirect modulatory effects [8, 66, 67]. The physiological role of GABAergic currents in the intrinsic cardiac ganglion and their impact on heart rate control need further investigation. However, in rat GABA-evoked currents have been measured from intrinsic cardiac neurons, but the current amplitude declined with age suggesting a role of GABA_A_ receptors in the development of the rat heart [65]. The intrinsic cardiac neurons, or intrinsic cardiac ganglion, consist of both parasympathetic cholinergic and sympathetic adrenergic postganglionic neurons that receive input from the parasympathetic preganglionic neurons in the brainstem and the preganglionic sympathetic neurons found in the spinal cord. From here these neurons project to the sinoatrial node. This classical view of the autonomic ganglion functioning only as a passive relay station from the central nervous system to the pacemaker cells of the heart is too simple, because both interneurons and afferent neurons are also found in the intracardiac ganglion. This allows sensory information about the chemical and mechanical state of the heart to be signalled to other neurons within the intrinsic cardiac ganglion. This integration of signals from both cardiac and extracardiac afferents and how they interact with the cardiac adrenergic and cholinergic motorneurons are important for regulating cardiac function (for review see [16]). 

**Figure 3:**
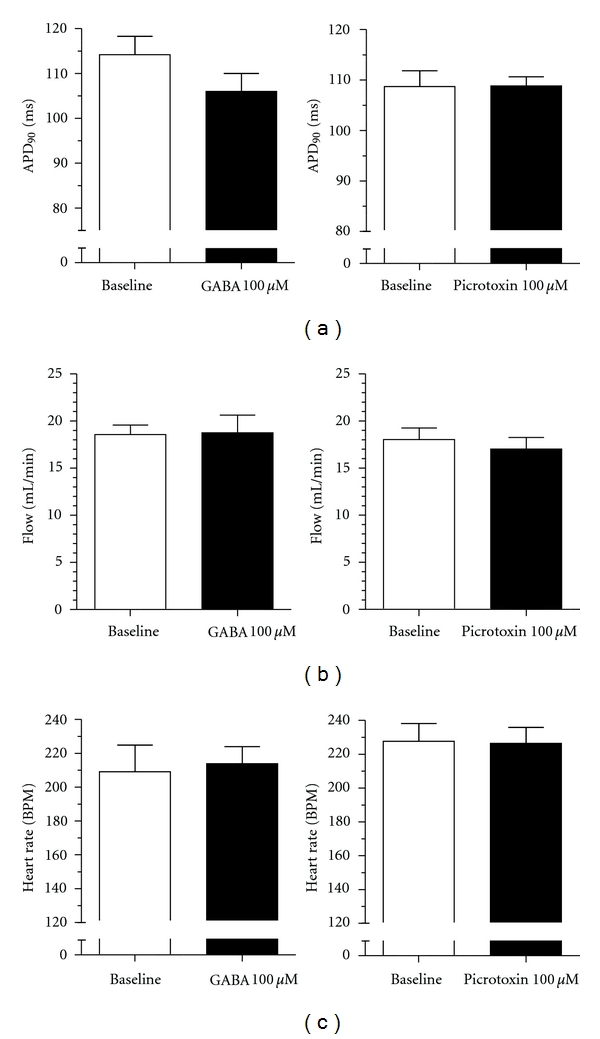
Effect of GABA 100 *μ*M (left column) or picrotoxin 100 *μ*M (right column) on isolated retrograde perfused female guinea pig hearts. Hearts were excised, mounted in a Langendorff apparatus, instrumented, and perfused with krebs-henseleit solution at a constant pressure of 60 mmHg as previously described [29]. Hearts were left to stabilize for a minimum of 30 min. After 20 min baseline recordings, where flow and heart rate was monitored, the hearts were paced from the right atrium for 2 min at 240 BPM. This protocol was repeated in the presence of GABA 100 *μ*M or picrotoxin 100 *μ*M (*n* = 5; GABA: 677 ± 99 g; Picrotoxin: 672 ± 100 g). GABA 100 *μ*M or picrotoxin 100 *μ*M produced no significant effect on action potential duration (a), flow (b), and heart rate (c).

The isolated perfused heart is often used for cardiovascular safety pharmacology. The importance of GABA_A_ receptors in this preparation has been investigated. In rats, adding GABA to the perfusion solution resulted in a dose-dependent reduction in heart rate [68]. We did not observe bradycardia in the isolated perfused guinea pig heart at a GABA concentration, which was found to affect the heart rate in rat, nor was there any significant effect of GABA on action potential duration or coronary flow. Likewise blocking GABA_A_ receptors by administration of picrotoxin did not produce any significant effects (Figure 3). 

**Figure 4:**
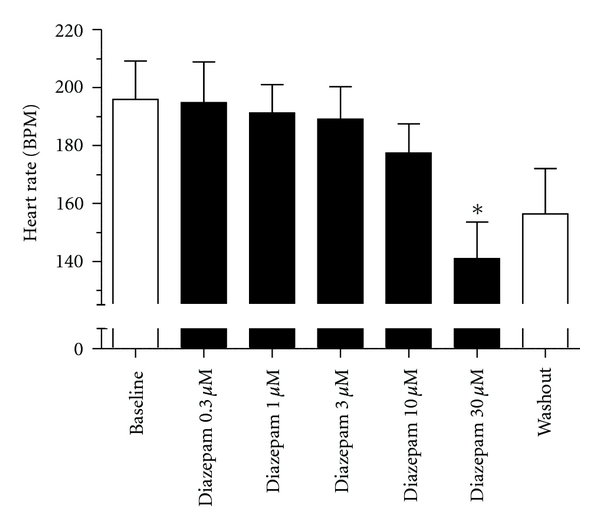
Effect of diazepam on isolated retrograde perfused female guinea pig hearts (868 ± 45 g). After a minimum of 30 min stabilization, the hearts underwent 20 min of baseline recording. Subsequently the hearts were exposed to diazepam at increasing concentrations in 20 min intervals. Diazepam induced a dose-dependent reduction of heart rate and significantly shortened the heart rate at 30 *μ*M (*n* = 5).

**Figure 5:**
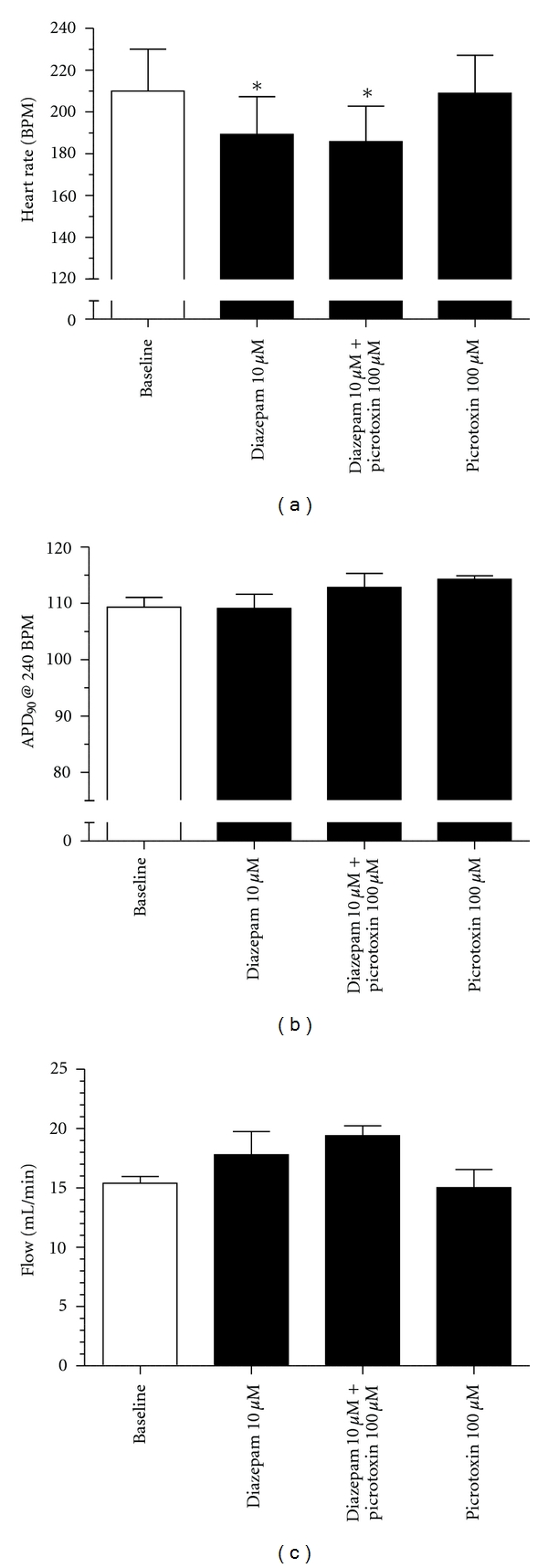
The effect of diazepam (10 *μ*M), diazepam (10 *μ*M) + picrotoxin (100 *μ*M) (co-administration), and picrotoxin (100 *μ*M) on heart rate (a), action potential duration (b), and coronary flow (c). Hearts were left to stabilize for a minimum of 30 min. 20 min of baseline recordings were performed, where heart rate and flow were monitored. The hearts were then paced from the right atrium at 240 BPM for 2 min in order to measure the action potential duration at a fixed heart rate. This (20+2) protocol was repeated for the different drugs investigated (*n* = 5; *P ≤ 0.05 (1-way ANOVA); (715 ± 129 g)).

In order to obtain a more thorough overview of possible GABAergic effects on isolated heart we also tested the effect of positive allosteric modulation. In contrast to agonist application in the form of GABA, we found that diazepam produced a concentration-dependent reduction in heart rate (Figure 4). This effect on heart rate was however not prevented by coadministration of picrotoxin (Figure 5(a)), suggesting that GABA_A_ receptors are not involved in the bradycardiac effect. Another study found that diazepam produced a negative inotropic response in isolated perfused guinea pig hearts that was not prevented by cotreatment with GABA_A_ or GABA_B_ receptor antagonists [69]. Further, diazepam application had no effect on action potential duration (APD) addressed by APD_90_ values or on coronary flow (Figures 5(b) and 5(c)). Diazepam has been found to inhibit the cardiac calcium channel recorded from isolated guinea pig cardiomyocytes [69], and to inhibit recombinant L-type voltage-gated calcium channels [70]. The reduced calcium influx can explain the observed GABA_A_ receptor-independent effects of diazepam on heart rate and contractility. However, it should be noted that the concentrations needed to produce calcium channel block, and the bradycardia and negative inotropy observed in the isolated guinea pig hearts are many folds above the therapeutic free plasma concentration (~0.2 *μ*M) and would so only be encountered during overdose.

## 6. Conclusion

This paper focused on the effect of GABA on the cardiac vagal neurons and on the isolated heart. It is important to recognize that regulation of heart rate not only involves these parts, but is part of a complex integrated system involving neurons located from the level of the insular cortex to the level of the heart [16]. Because GABA is the main inhibitory neurotransmitter in the central nervous system, the cardiac effects of modulating GABA_A_ receptor activity, especially with respect to GABA_A_ receptor subtype selective compounds, are hard to predict and require careful investigation. In this respect it will be interesting to obtain more knowledge about the GABA_A_ receptor subunit composition in the neuronal pathways involved in heart rate control. Considering the profound effects of anaesthetics on the GABAergic system, and on neuronal pathways involved in heart rate control, it argues for the use of conscious freely moving animals at early stages during cardiovascular safety pharmacology profiling of novel compounds targeting the GABA_A_ receptor. Such cardiovascular safety pharmacology investigations could well be combined with behavioural assessment [71]. This will aid in interpretation of drug-induced effects and increase the amount of data generated per animal, thereby reducing the number of animals used.
